# Supplementary figures and images for: Open source 3D phenotyping of chickpea plant architecture across plant development
Source: Plant Methods. 2021 Sep 16;17:95. doi: 10.1186/s13007-021-00795-6 (PMC8444385; doi:10.1186/s13007-021-00795-6)

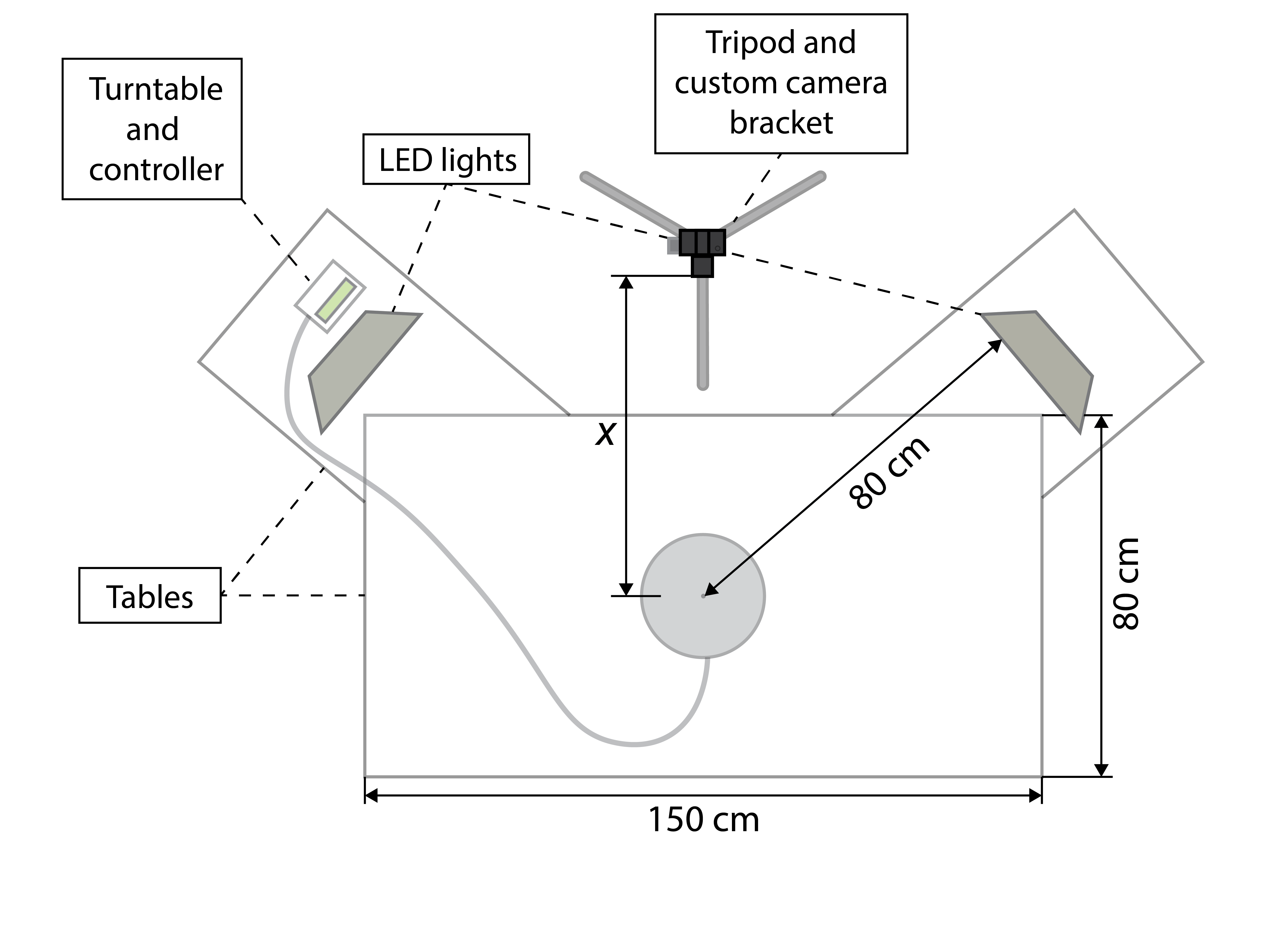

Supplement: Supplementary file 11 — Additional file 11. Top down diagram of the laboratory imaging set up. The tripod was moved back from the table (distance x) as the plants grew. [file 13007_2021_795_MOESM11_ESM.png]

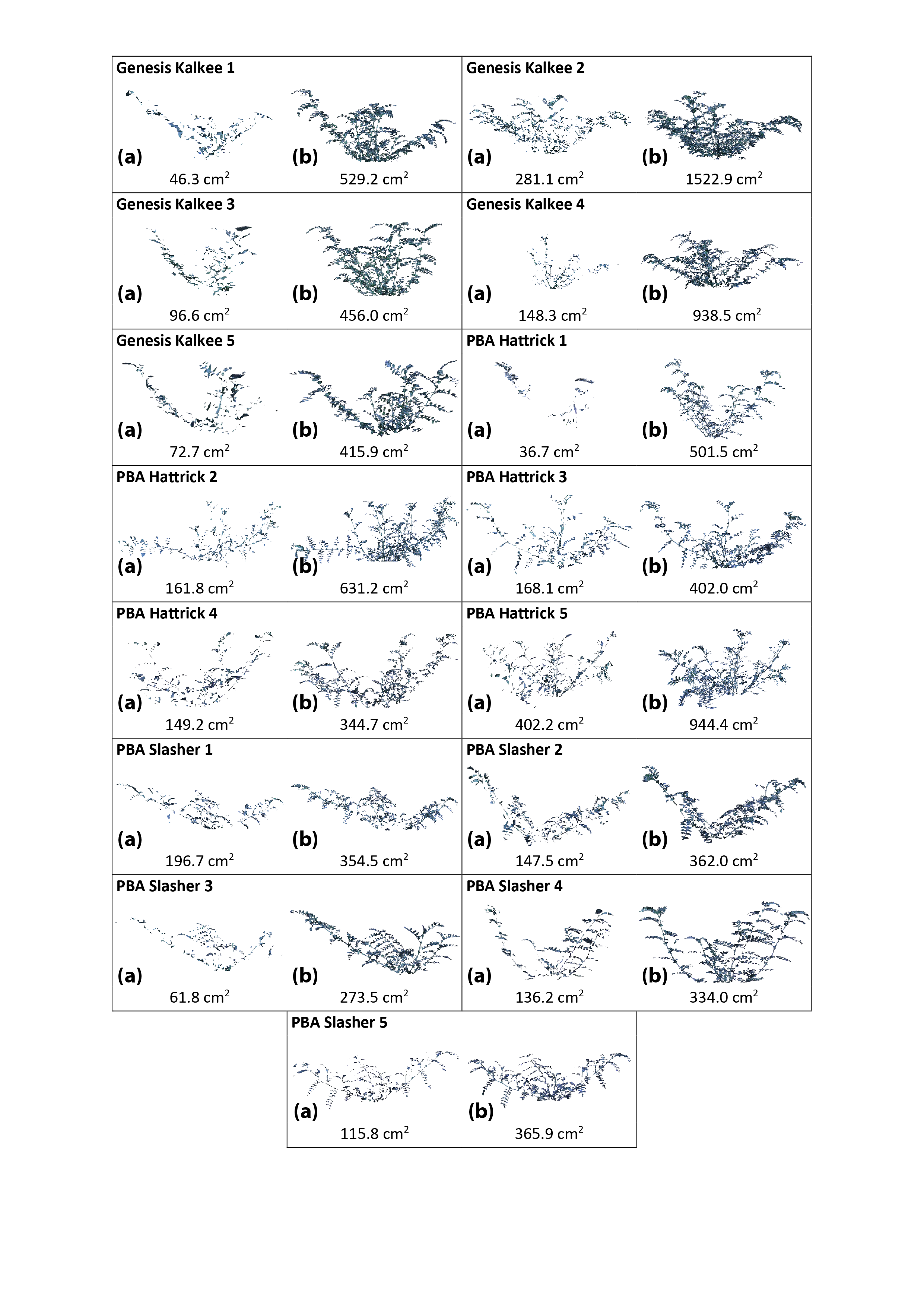

Supplement: Supplementary file 12 — Additional file 12. Comparison of 3D reconstructions generated using wheat and chickpea parameters in VisualSFM. Week 5 plants were reconstructed (a) using 40 images and the reconstruction parameters for wheat as per Burgess et al. [13], and (b) using the parameters fine-tuned to work with chickpea plants of various sizes. The numbers below each image refer to the estimated surface area of each reconstruction. [file 13007_2021_795_MOESM12_ESM.png]

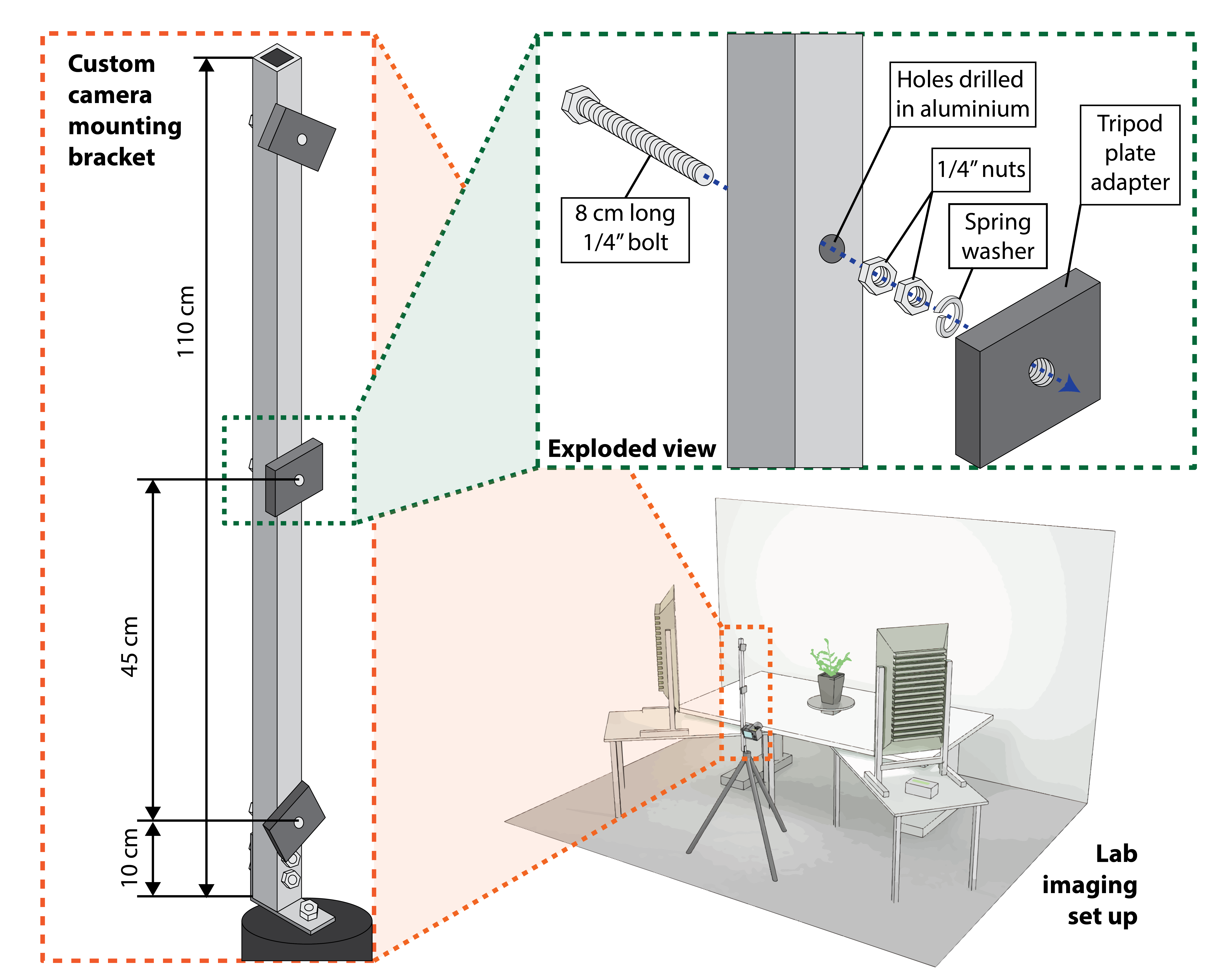

Supplement: Supplementary file 13 — Additional file 13: Figure S3. Schematic diagram of the custom camera mounting bracket. [file 13007_2021_795_MOESM13_ESM.png]

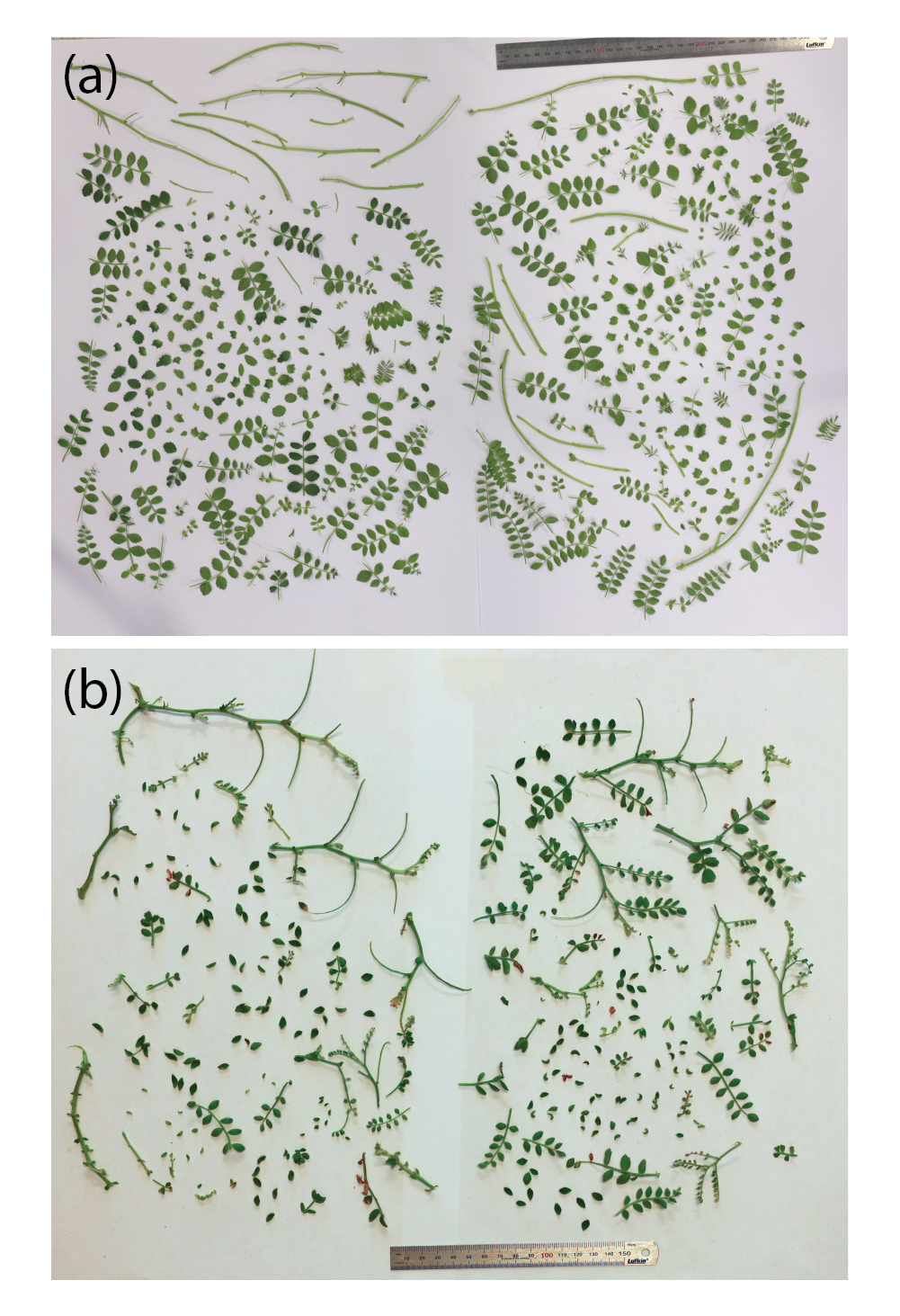

Supplement: Supplementary file 14 — Additional file 14: Figure S4. Two representative images of a harvested chickpea plants used for ground truthing measurements. (a) Is the commercial chickpea cultivar Genesis Kalkee and (b) is the breeding line PUSA76. [file 13007_2021_795_MOESM14_ESM.png]

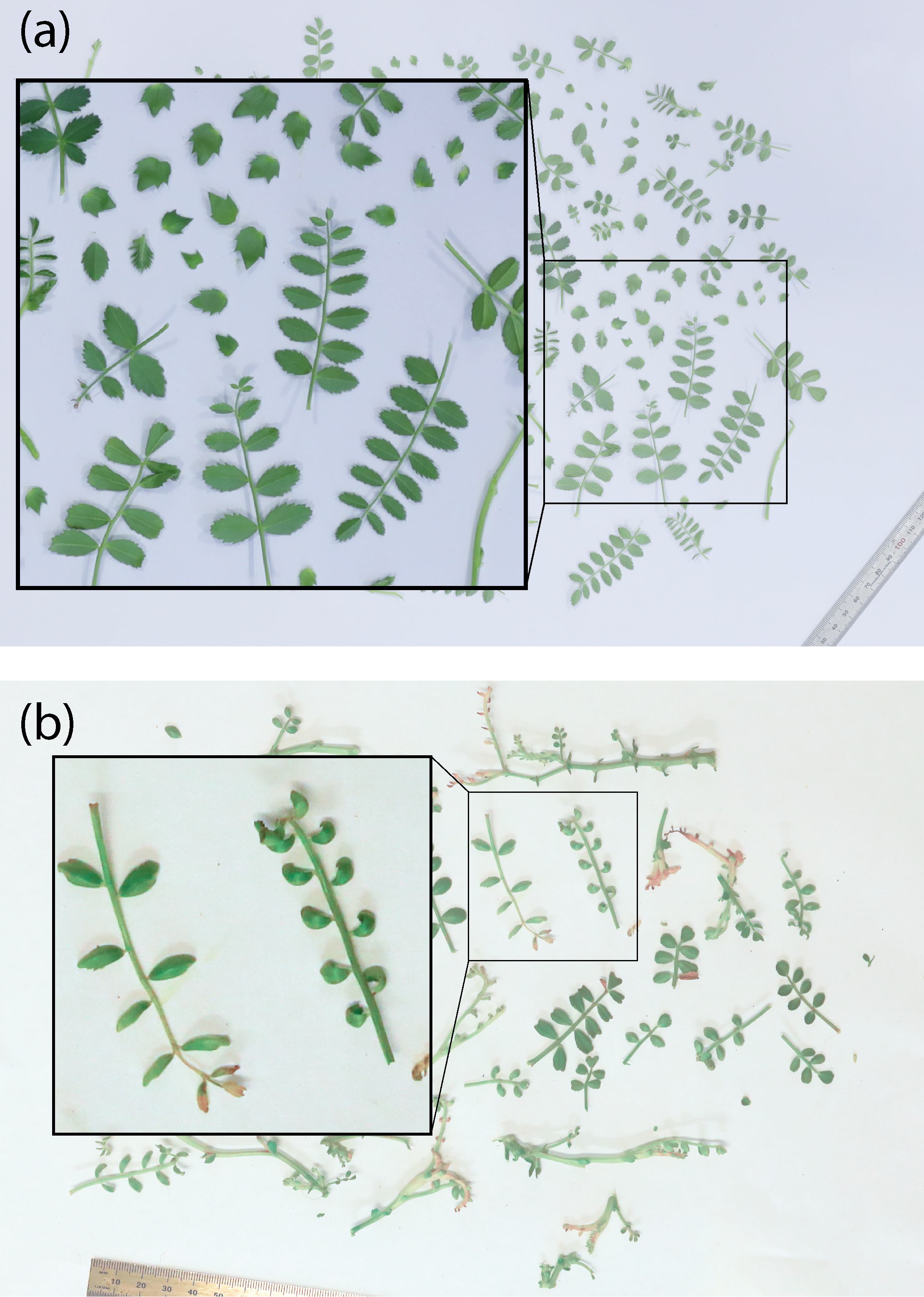

Supplement: Supplementary file 15 — Additional file 15: Figure S5. An example of curled leaves of the outdoor-grown chickpea breeding lines. (a) Shows a representative plant of the commercial cultivar, the inset figure shows that most leaves were flat. (b) Shows a representative plant of breeding line SonSla, the inset figure shows that many leaves were curled or folded to a certain degree. [file 13007_2021_795_MOESM15_ESM.png]

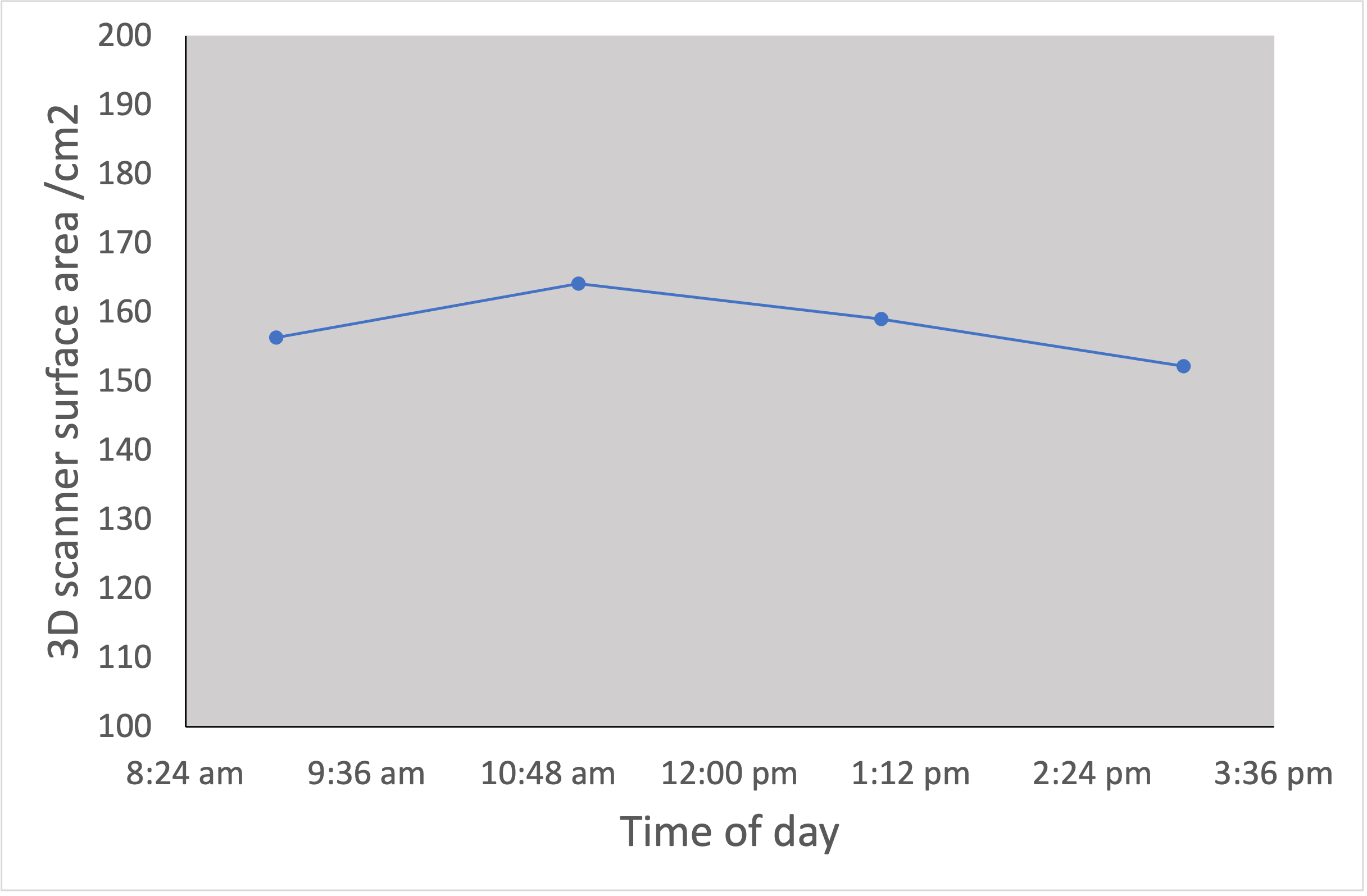

Supplement: Supplementary file 16 — Additional file 16: Figure S6. Diurnal measurements of plant surface area using the 3D scanner. A single plant was scanned four times between 9 a.m. and 3 p.m. to ensure there was not substantial variation in surface area estimates due to leaf movement/closure across the course of a day. [file 13007_2021_795_MOESM16_ESM.png]
