# Supplementary material for: Open source 3D phenotyping of chickpea plant architecture across plant development
Source: Plant Methods. 2021 Sep 16;17:95. doi: 10.1186/s13007-021-00795-6 (PMC8444385; doi:10.1186/s13007-021-00795-6)
Supplement: Supplementary file 17 — Additional file 17: Table S1. Genotype specific regressions for validation measurement. Significance values refer to an ANOVA run to determine whether there were differences across genotypes. Table S2. Chickpea genotypes used to validate the 3D scanner. These lines were chosen based on their contrasting canopy heights, growth habits and growth rates. Note that some information is lacking for breeding lines. Table S3. Comparison of side projected area estimates from 2D image analysis. [file 13007_2021_795_MOESM17_ESM.docx]

**Additional file 17**

**Table S1.** Genotype specific regressions for validation measurement. Significance values refer to an ANOVA run to determine whether there were differences across genotypes.

| **Genotype** | **Regression** | **R^2^** | **n** |
| --- | --- | --- | --- |
| **Height validation^n.s.^** |  |  |  |
| Genesis Kalkee | y = 0.993x | 0.999 | 45 |
| PBA Hattrick | y = 0.970x | 0.999 | 45 |
| PBA Slasher | y = 0.963x | 0.999 | 45 |
| ICC5878 | y = 0.941x | 0.996 | 12 |
| SonSla | y = 0.951x | 0.996 | 12 |
| PUSA76 | y = 0.975x | 0.998 | 12 |
| **Area validation*** |  |  |  |
| Genesis Kalkee | y = 0.986x | 0.997 | 20 |
| PBA Hattrick | y = 1.072x | 0.988 | 20 |
| PBA Slasher | y = 0.941x | 0.994 | 20 |
| ICC5878 | y = 1.122x | 0.980 | 12 |
| SonSla | y = 1.137x | 0.979 | 12 |
| PUSA76 | y = 1.167x | 0.993 | 12 |
| **Significance** |  |  |  |
| ^n.s.^ = P > 0.05; * = P < 0.05; ** = P < 0.01; *** = P <0.001. | | | |
|  | | | |

**Table S2.** Chickpea genotypes used to validate the 3D scanner. These lines were chosen based on their contrasting canopy heights, growth habits and growth rates. Note that some information is lacking for breeding lines.

| **Genotype** | **Type** | **Plant height** | **Growth habit** | **Vigour** |
| --- | --- | --- | --- | --- |
| Genesis Kalkee | Kabuli | Medium/tall | Erect | Mid |
| PBA Hattrick | Desi | Tall | Erect | Mid |
| PBA Slasher | Desi | Medium/short | Spreading | Late |
| ICC5878 | Desi | Short | - | - |
| SonSla | Desi | - | Prostrate | - |
| PUSA76 | Desi | - | - | Early |

**Table S3.** Comparison of side projected area estimates from 2D image analysis.

| **Genotype** | **Image pair** | **Side projected area (cm^2^)** | | **Difference**  **(cm^2^)** | **Difference**  **(%)** |
| --- | --- | --- | --- | --- | --- |
|  |  | **Front** | **Side** |  |  |
| Genesis Kalkee | 1 | 102.2 | 103.6 | 1.5 | 1.4 |
|  | 2 | 245.3 | 238.1 | 7.2 | 3.0 |
|  | 3 | 104.9 | 109.1 | 4.1 | 3.9 |
|  | 4 | 204.2 | 207.6 | 3.4 | 1.6 |
|  | 5 | 81.1 | 88.6 | 7.5 | 8.8 |
| PBA Hattrick | 1 | 112.1 | 133.3 | 21.2 | 17.3 |
|  | 2 | 169.1 | 177.8 | 8.8 | 5.1 |
|  | 3 | 87.3 | 86.2 | 1.1 | 1.3 |
|  | 4 | 120.9 | 111.3 | 9.6 | 8.3 |
|  | 5 | 224.8 | 202.4 | 22.4 | 10.5 |
| PBA Slasher | 1 | 119.9 | 108.3 | 11.7 | 10.2 |
|  | 2 | 63.4 | 82.3 | 18.9 | 26.0 |
|  | 3 | 69.1 | 70.8 | 1.7 | 2.4 |
|  | 4 | 59.3 | 55.0 | 4.3 | 7.5 |
|  | 5 | 58.1 | 43.5 | 14.6 | 28.7 |
